# Supplementary material for: AKT1 mediates multiple phosphorylation events that functionally promote HSF1 activation
Source: FEBS J. 2022 Feb 11;289(13):3876–93. doi: 10.1111/febs.16375 (PMC9309721; doi:10.1111/febs.16375)
Supplement: Supplementary file 1 — Fig. S1. HSF1 protein stability is not affected by AKT1 presence. Fig. S2. Serine to Glutamate Mutation does not result in a Phospho‐mimic for HSF1 phosphorylation sites. Fig. S3. HSF1 activity in the presence of CDK9 and TFIIB is reduced with T527A mutation. Table S1. AKT1 has a unique phosphorylation pattern on the HSF1 protein. [file FEBS-289-3876-s001.zip › febs16375-sup-0001-Supinfo.pdf]

FEBS PRESS science publishing by scientists

WILEY

The FEBS Journal

www.febsjournal.org

AKT1 mediates multiple phosphorylation events that functionally promote HSF1 activation

Wen-Cheng Lu, Ramsey Omari, Haimanti Ray, John Wang, Imade Williams, Curteisha Jacobs, Natasha Hockaden, Matthew L. Bochman and Richard L. Carpenter

DOI: 10.1111/febs.16375

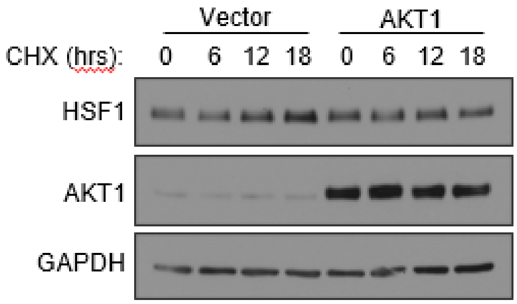

Supplemental Figure 1: HSF1 protein stability is not affected by AKT1 presence. HEK-293 cells were transfected with either an empty vector or AKT1 followed by treatment with cycloheximide (10 ug/mL) for up to 18 hrs. Cells were then lysed and total protein subjected to immunoblotting with indicated antibodies. HSF1 protein stability was not altered in the presence of AKT1.

Kinase	HSF1 Peptide	Peptide Start	P-site
AKT1	SVTKLL**pT**DVQLM	136	T142
	**pS**LEHVHGSGPY	230	S230
	TGSEPPKAKDP**pT**VS	516	T527
AKT2	**pS**APSPAYSSSSLY	241	S241
	TDARGHTDTEGRPP**pS**PPPTSTPEKCL	349	S363
P38	**pS**APSPAYSSSSLY	241	S241
	TDARGHTDTEGRPP**pS**PPPTSTPEKCL	349	S363
mTORC1	MLNDSGSAH**pS**MPKYSRQF	212	S221
	**pS**APSPAYSSSSLY	241	S241
	IDSILRESEPAPA**pS**VTAL	331	S344
MEK1	TGSEPPKAKDP**pT**VS	516	T527
DYRK2	SLEHVHGSGPY**pS**APSPAYSSSSL	230	S241
	TDARGHTDTEGRPP**pS**PPPTSTPEKCLSVA	349	S363

Supplemental Table 1: AKT1 has a unique phosphorylation pattern on the HSF1 protein. Purified HSF1 protein were incubated with the indicated kinases for 2 hrs at 30°C. Proteins were then subjected to mass spectrometry as described in the materials and methods to identify HSF1 phosphorylated residues. HSF1 phosphorylated peptides are presented along with the corresponding phosphorylated site for each kinase tested.

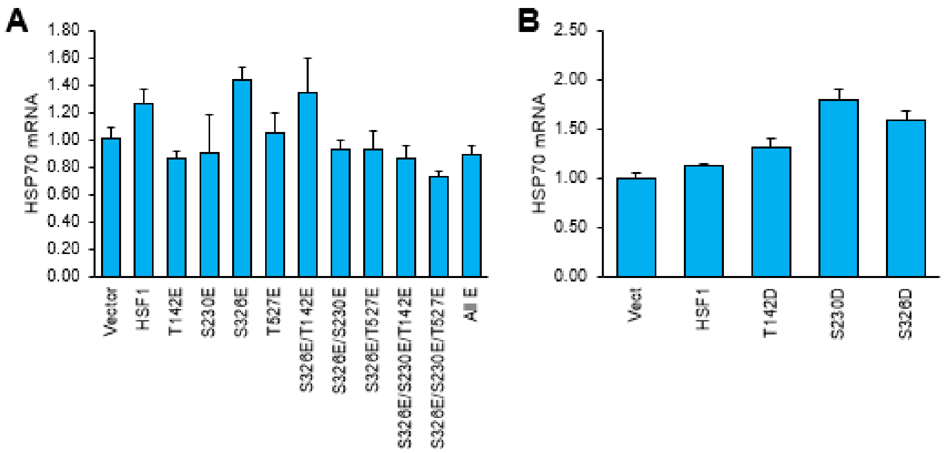

Supplemental Figure 2: Serine to Glutamate Mutation does not result in a Phospho-mimic for HSF1 phosphorylation sites. A-B) Wild-type HSF1 underwent mutagenesis at the indicated residues. An empty vector, wild-type HSF1, and each of these mutants were expressed in HEK-293 cells. Total RNA was subjected to RT-qPCR for HSP70, an HSF1 target gene. None of these phospho-mimic constructs were able to consistently increase HSF1 activity over the wild-type suggesting they do not sufficiently mimic these residues being phosphorylated.

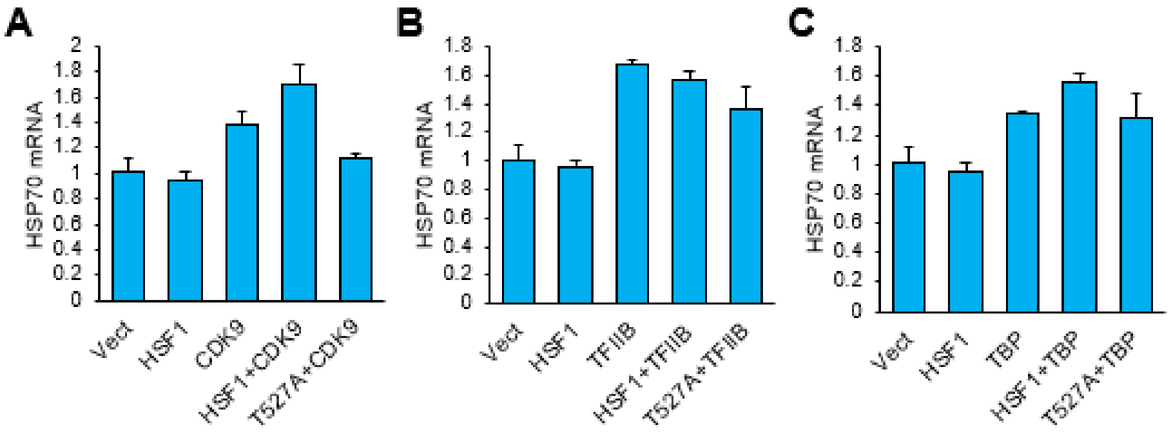

Supplemental Figure 3: HSF1 activity in the presence of CDK9 and TFIIB is reduced with T527A mutation. Wild-type HSF1 was mutated to give T527A mutation. Empty vector, wild-type HSF1, or T527A were expressed with or without CDK9 (A), TFIIB (B), or TBP (C). Total RNA was subjected to RT-qPCR for HSP70, a direct target gene of HSF1. T527A mutation significantly reduced HSF1 activity in the presence of CDK9 and TFIIB.
